# Inhibition of Autophagy Does Not Re-Sensitize Acute Myeloid Leukemia Cells Resistant to Cytarabine

**DOI:** 10.3390/ijms22052337

**Published:** 2021-02-26

**Authors:** Nienke Visser, Harm Jan Lourens, Gerwin Huls, Edwin Bremer, Valerie R. Wiersma

**Affiliations:** Department of Hematology, Cancer Research Center Groningen, University Medical Center Groningen, University of Groningen, 9713 GZ Groningen, The Netherlands; n.visser@umcg.nl (N.V.); h.j.lourens@umcg.nl (H.J.L.); g.huls@umcg.nl (G.H.); e.bremer@umcg.nl (E.B.)

**Keywords:** AML, autophagy, cytarabine, therapy resistance, autophagy inhibitors, chloroquine

## Abstract

Elevated activation of the autophagy pathway is currently thought to be one of the survival mechanisms allowing therapy-resistant cancer cells to escape elimination, including for cytarabine (AraC)-resistant acute myeloid leukemia (AML) patients. Consequently, the use of autophagy inhibitors such as chloroquine (CQ) is being explored for the re-sensitization of AraC-resistant cells. In our study, no difference in the activity of the autophagy pathway was detected when comparing AraC-Res AML cell lines to parental AraC-sensitive AML cell lines. Furthermore, treatment with autophagy inhibitors CQ, 3-Methyladenine (3-MA), and bafilomycin A1 (BafA1) did not re-sensitize AraC-Res AML cell lines to AraC treatment. However, in parental AraC-sensitive AML cells, treatment with AraC did activate autophagy and, correspondingly, combination of AraC with autophagy inhibitors strongly reduced cell viability. Notably, the combination of these drugs also yielded the highest level of cell death in a panel of patient-derived AML samples even though not being additive. Furthermore, there was no difference in the cytotoxic effect of autophagy inhibition during AraC treatment in matched de novo and relapse samples with differential sensitivity to AraC. Thus, inhibition of autophagy may improve AraC efficacy in AML patients, but does not seem warranted for the treatment of AML patients that have relapsed with AraC-resistant disease.

## 1. Introduction

Current standard therapy for acute myeloid leukemia (AML) patients is a combination of cytarabine (AraC) with either daunorubicin or idarubicin [1]. This intensive chemotherapy regimen induces complete remission in ~65% of newly diagnosed AML patients, especially in patients under the age of 60 [2]. However, within the first 1–2 years, ~66% of these responders will eventually relapse predominantly due to development of resistance to AraC treatment. Hence, the search for strategies that prevent or overcome AraC resistance is of importance to improve the prognosis of AML patients.

One of the mechanisms described to cause and sustain AraC resistance in AML is by the activation of the autophagy pathway, which is a pathway that regulates the recycling of damaged and superfluous cellular content during both homeostasis and stress as reviewed in Reference [3]. In brief, during macro-autophagy (hereafter called autophagy), the activation of two ubiquitin-like conjugation systems comprising the sequential activation of autophagy-related (ATG) proteins and lipidation of microtubule-associated protein 1A/1B-light chain 3 (LC3) leads to the formation of autophagosomes. Subsequently, proteins are targeted for the degradation of these autophagosomes by cargo proteins such as p62. In the final stage, the mature autophagosomes fuse with lysosomes, whereby the content of the auto-phagolysosome is degraded by lysosomal proteases.

In previous studies, AML cell lines REH and HL-60 cells activated the autophagy pathway during AraC treatment as a survival mechanism [4]. Furthermore, U-937 and AML-2 cells resistant to AraC had an increased basal activity of the autophagy pathway compared to parental cell lines [5,6]. In line with this data, AraC resistant U-937 and AML-2 cells were more sensitive to the autophagy inhibitor chloroquine (CQ) than parental cell lines [5,6]. Furthermore, using either chemical inhibitors or by downregulation of autophagy genes, the inhibition of autophagy increased the cytotoxic effect of AraC in either parental AML cell lines REH, HL-60, and U-937 [4,6,7] or AraC resistant cell lines U-937 and AML-2 [5,6]. Collectively, this data argues for the incorporation of autophagy inhibitors into the treatment of AML, even though a thorough definitive assessment of the relevance of autophagy in AML is lacking, especially in relevant AML patient samples. Based on our knowledge, the efficacy of autophagy inhibition in combination with AraC has predominantly been tested in cell lines and has not been evaluated in a substantial number of AML patient samples (only two samples in Reference [5]). Moreover, current studies investigated the activity of the autophagy pathway either during AraC treatment in parental cells [4,7] or under basal circumstances in established AraC resistant cell lines compared to parental cell lines [5,6], whereas a thorough analysis of both aspects within one and the same study is lacking.

In the current study, we analyzed the activity of the autophagy pathway in parental (Par) versus AraC resistant (AraC-Res) cell lines in a basal state as well as upon AraC treatment. Furthermore, we evaluated the effect of autophagy inhibition during AraC treatment in cell lines as well as patient-derived AML cells, including matched de novo and relapse samples. The autophagic flux was not elevated in AraC-Res cell lines compared to parental cells, and also matched de novo and relapse AML patient samples had comparable levels of autophagy. In line with this data, AraC-Res cell lines could not be re-sensitized for AraC using autophagy inhibitors. In contrast, in AraC-sensitive parental cells, a clear, additive cytotoxic effect was detected upon combination of AraC with autophagy inhibitors. However, in both de novo and relapse AML patient samples, there was no additive, cytotoxic effect of co-treatment with CQ and AraC, even though the highest levels of cell death were achieved when combining these drugs. Together, this data indicates that the inhibition of autophagy does not re-sensitize AraC-Res AML cells for AraC and that the inhibition of autophagy using CQ, even though having an effect in cell lines, likely does not impact the efficacy of AraC in relapsed AML patients.

## 2. Results

### 2.1. Generation of Cytarabine Resistant Cell Lines

**Figure 1:**
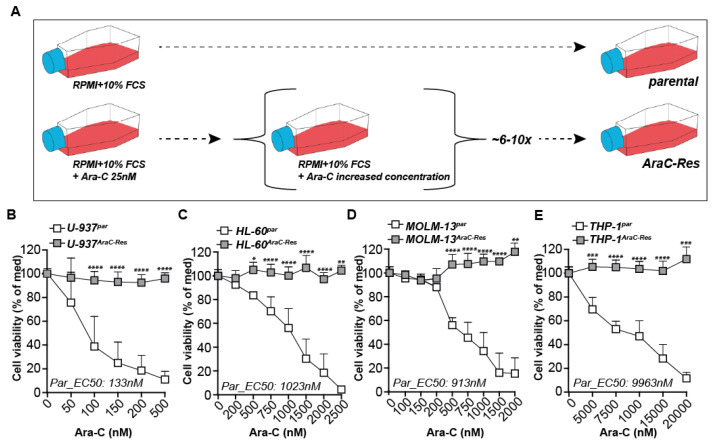
Generation of cytarabine resistant cell lines. (**A**) Acute myeloid leukemia (AML) cell lines stably resistant to cytarabine (AraC-Res) were generated by culturing AML cell lines under gradually increasing doses of AraC for up to a year (~6–10 times increase in AraC dose). (**B**–**E**) Cell viability of all cell line pairs upon treatment with a dose range of AraC as measured by MTS assays after 72 h of incubation (at least *n* = 5). Significance was tested using students’ *t*-test. *p* values are indicated as: **** *p* < 0.0001, *** *p* < 0.001, ** *p* < 0.01, and * *p* < 0.05.

AML cell lines stably resistant to cytarabine (AraC-Res) were obtained by culturing under gradually increasing doses of AraC for up to a year (Figure 1A). The dose of AraC was doubled when the growth of the cells under AraC pressure equaled the proliferation speed of the parental cells. Subsequent treatment of pairs of the parental and AraC-res cell lines with AraC yielded a clear dose-dependent decrease in cell viability for the parental U-937 (Figure 1B), HL-60 (Figure 1C), MOLM-13 (Figure 1D), and THP-1 cells (Figure 1E). However, similar treatment of the AraC-Res clones did not significantly reduce cell death even at the high doses tested for any of the cell lines (Figure 1B–E). AraC-Res cell lines remained resistant without AraC pressure for extended periods of testing, with testing halted after four months (Supplementary Figure S1A–H).

### 2.2. The Expression of General Autophagy Genes and Proteins Is Not Uniformly Elevated in AraC Resistant AML Cells

**Figure 2:**
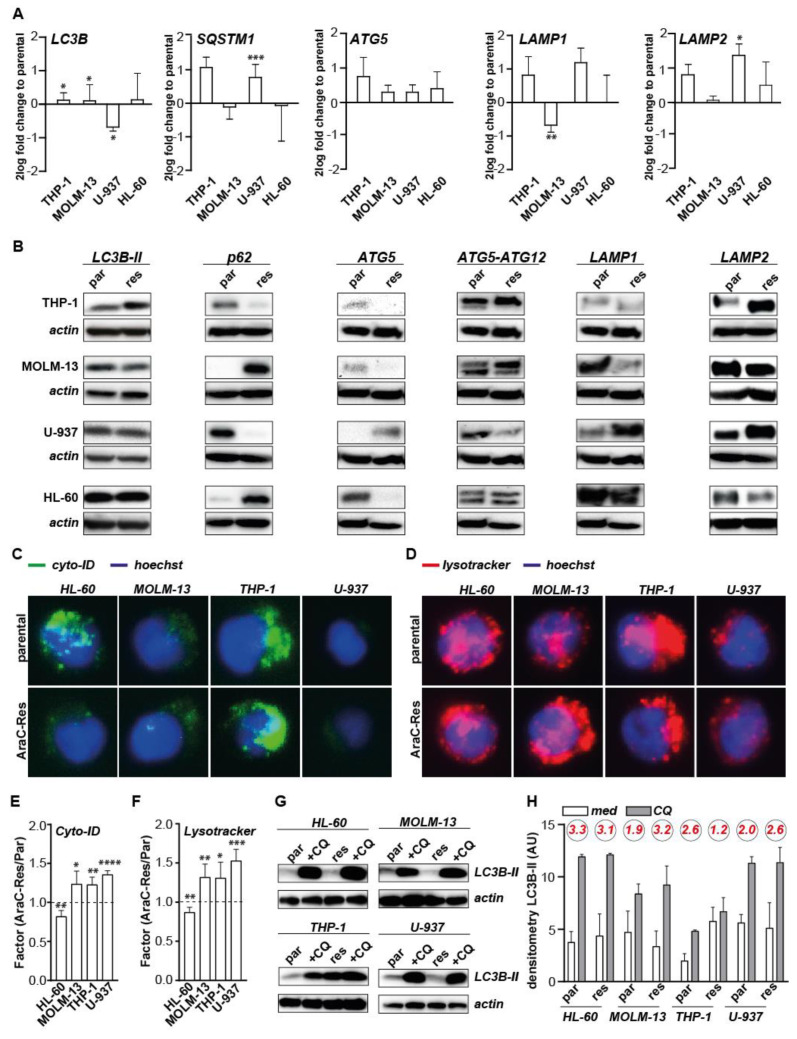
Expression of general autophagy genes and proteins is not elevated in AraC resistant AML cells. (**A**) mRNA expression levels of autophagy genes measured in all cell line pairs and presented as a log fold change of the AraC-Res cell line compared to the parental cell line (*n* = 3). (**B**) Representative protein expression levels of autophagy-related proteins (LC3B-II (14 kDa), p62 (62 kDa), ATG5 (32 kDa), ATG5-ATG12 (55 kDa), LAMP1 (130 kDa), LAMP2 (130 kDa), and loading control beta-actin (42 kDa) in parental and AraC-Res cell lines as determined by Western blot (*n* = 3). (**C**) Representative fluorescent pictures of parental and AraC-Res cell lines stained for basal autophagosomal content using Cyto-ID, captured at 40× magnification. (**D**) As in (**C**) but stained for basal lysosomal content using lysotracker. (**E**,**F**) The difference in basal cyto-ID and lysotracker signal between AraC-Res and parental cell lines (depicted as a factor, AraC-Res/parental) as measured using flow cytometry (*n* = 4). (**G**) Representative Western blots showing the basal autophagic flux in parental versus AraC-Res cell lines using chloroquine (CQ, 50 μM for 6 h) (*n* = 2). (**H**) Densitometry measurements of the experiment as shown in (**G**), showing the factor increase in LC3B-II in CQ treated versus untreated cells in red (*n* = 2). Significance was tested using a student’s t-test. *p* values are indicated as: **** *p* < 0.0001, *** *p* < 0.001, ** *p* < 0.01, and * *p* < 0.05.

Since, in several previous reports, autophagy upregulation was reported to contribute to AraC resistance in AML [5,6], the AraC-Res AML cell line panel was first evaluated for expression levels of key autophagy genes at the mRNA level. Compared to parental cell lines, the mRNA expression level of the key autophagy gene *LC3B* slightly increased in two out of four of the AraC-Res cell lines, but even decreased in U-937^AraC-Res^ cells (Figure 2A). Furthermore, mRNA expression levels of *SQSTM1* (p62) and *LAMP1* increased in U-937^AraC-Res^ and THP-1^AraC-Res^ cells, which was only significant for *SQSTM1* in U-937 (Figure 2A). Furthermore, *LAMP1* mRNA levels only significantly changed in MOLM-13^AraC-Res^ cells in which the expression was decreased (Figure 2A). *LAMP2* mRNA levels did increase in three out of four cell lines, which was only significant for U-937, whereas *ATG5* expression was uniformly, but not significantly, increased in AraC-Res cell lines compared to parental cells (Figure 2A). Protein expression levels of LC3B-II, LAMP1, and LAMP2 closely followed the expression pattern of mRNA, with all three proteins being both upregulated and downregulated in AraC-Res versus parental cell lines (Figure 2B). Strikingly, p62 protein levels were opposite of mRNA levels, possibly due to active degradation of p62 during execution of autophagy. Free ATG5 protein levels (detected at 32 kDa) mirrored mRNA levels in three out of four AraC-Res cell lines. In contrast, no clear and/or uniform difference between parental and AraC-Res cell lines were detected for the ATG5-ATG12 complex (detected at 55 kDa), which is formed during the activation of autophagy. Thus, based on both mRNA and protein expression levels of key autophagy genes, there is no clear uniform induction of the autophagy pathway in AraC-Res versus parental cell lines.

### 2.3. The Basal Level of Autophagic Flux Is Not Elevated in AraC Resistant Cells Compared to Parental Cells

To further investigate the autophagy pathway in parental versus AraC-Res cell lines, organelle-specific fluorescent dyes were used to stain the autophagosomes and lysosomes. The strength of cyto-ID fluorescence, staining autophagosomes, and strength of lysotracker fluorescence, staining lysosomes, clearly differed between the various parental cell lines (Figure 2C,D), with the most prominent staining in THP-1 cells and almost undetectable staining in U-937 cells. However, no marked differences were detected between parental and Ara-C resistant pairs, with the exception of cyto-ID staining in HL-60 cells where staining intensity was reduced in AraC-Res cells. Similar results were obtained when staining was quantified by flow cytometry as fold change compared to parental cells (Figure 2E,F), with cyto-ID and lysotracker signal being lower in HL-60^AraC-Res^ and in all other cell line pairs being slightly higher in AraC-Res cell lines. However, higher intrinsic autophagosome and lysosome content does not necessarily resemble an increased activity of the autophagy pathway or ‘autophagic flux’. Therefore, the basal autophagic flux was determined using chloroquine (CQ), which is a lysomotropic agent that, by inhibiting lysosomal function, blocks the execution of autophagy, leading to the accumulation of LC3B-II that is actively formed during the activation of autophagy. These basal levels of autophagic flux did not clearly differ between parental and AraC-Res cell lines based on LC3B-II levels (Figure 2G). Furthermore, upon semi-quantitative densitometry, no differences in flux in HL-60 and U-937 cell line pairs, a slight increase in MOLM-13^AraC-Res^, and a reduction in THP-1^AraC-Res^ cells was detected when compared to parental cell lines (Figure 2H). Thus, based on mRNA and protein expression of autophagy genes and fluorescent staining of autophagosomes, lysosomes, and the basal autophagic flux, the basal activity of the autophagy pathway was not increased in AraC-Res compared to parental AML cells.

Of note, in literature, various other mechanisms that govern AraC resistance have been described, among others, the downregulation of the transporters that are responsible for either AraC uptake in the cell, e.g., equilibrative nucleoside transporter 1 and 2 (ENT1, ENT2) [8], or AraC transport outside the cell, e.g., Multi-Drug Resistance 1 (MDR1) [9], and the downregulation of deoxycytidine kinase (dCK), which is the kinase that converts AraC into its active form [10]. *ENT1* expression was strongly reduced in THP-1^AraC-Res^ cells compared to parental cells (Supplementary Figure S2A,B). Furthermore, *dCK* expression was reduced in all AraC-Res cell lines, with the strongest reduction in U-937 and HL-60 (Supplementary Figure S2C). *MDR1* was not detected in any of the cell lines (data not shown). In addition, elevated expression of the anti-apoptotic protein B-cell lymphoma 2 (BCL-2) has been implicated in AraC resistance [11,12]. However, only U-937^AraC-Res^ had slightly increased *BCL-2* expression levels compared to parental cells (Supplementary Figure S2D). Thus, AraC resistance in our cell line panel was not regulated by sustained elevated activity of the autophagy pathway, but rather by either downregulation of *ENT1* or *dCK* expression.

### 2.4. Autophagy Is Activated Upon AraC Treatment in Parental but Not in AraC Resistant Cells

**Figure 3:**
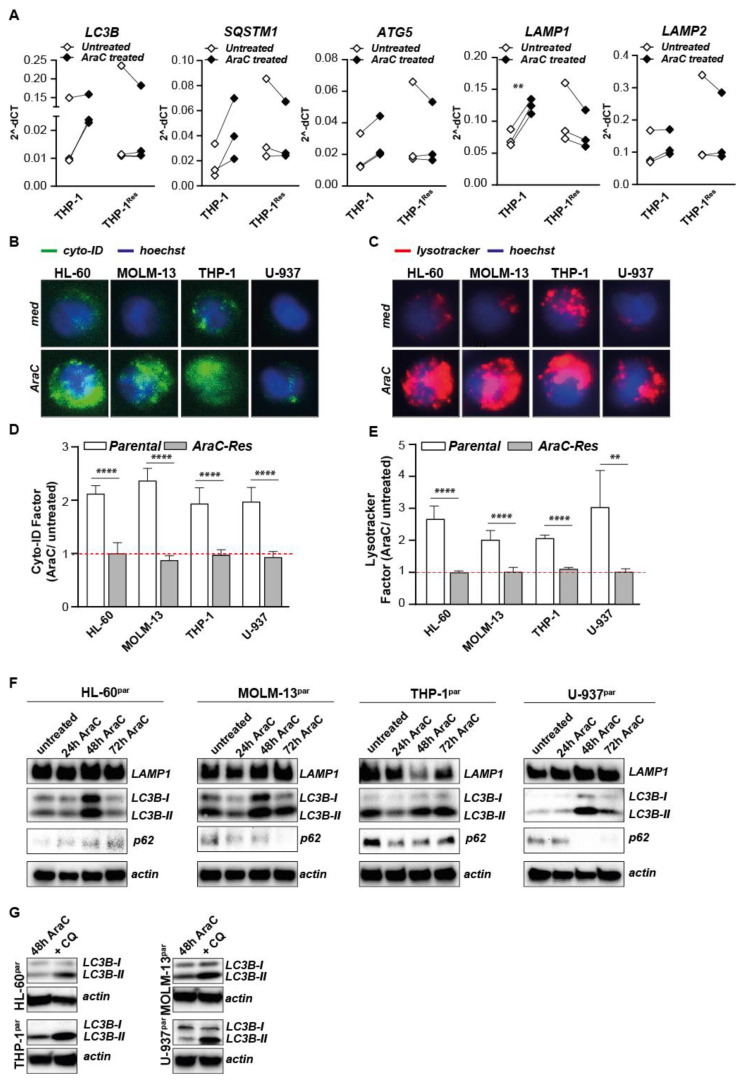
The autophagy pathway is activated upon AraC treatment in parental but not AraC resistant cells. (**A**) mRNA levels of *LC3B*, *SQSTM1*, *ATG5*, *LAMP1*, and *LAMP2* upon AraC treatment (24 h, 4000 nM AraC) in parental THP-1 and THP-1^AraC-Res^ cells (*n* = 3). (**B**,**C**) Representative fluorescent microscopy pictures showing the autophagosomal and lysosomal content in parental cell lines in untreated versus AraC treated cells, captured at 40× magnification. (**D**,**E**) As in (**B**,**C**) but quantified using flow cytometry Factor change in cyto-ID (**D**) and lysotracker (**E**) signal upon AraC treatment as determined by flow cytometry (*n* = 6 for parental cell lines and *n* = 3 for AraC-Res cell lines). (**F**) Representative Western blots showing the activity of the autophagy pathway during AraC treatment (24, 48, and 72 h) based on LC3B-II formation (*n* = 2). (**G**) Representative Western blots showing the activity of the autophagy pathway during AraC treatment (24, 48, and 72 h) based on p62 degradation (*n* = 2). In all experiments, a sub-lethal dose of AraC was used that reduced the cell viability with ~20%, i.e., 250 nM for HL-60 and MOLM-13, 100 nM for U-937 and 4000 nM for THP-1. Significance was tested using student’s *t*-test. *p* values are indicated as: **** *p* < 0.0001, ** *p* < 0.01.

Although the basal levels of autophagic flux are not elevated in AraC-Res cell lines, activation of autophagy during AraC treatment may still promote AML survival. mRNA levels of *LC3B, SQSTM1, ATG5, LAMP1*, and *LAMP2* are all increased for THP-1 and for almost all in the other cell lines upon treatment with AraC in parental cell lines, even though, in most cases, this increase is not significant (Supplementary Figure S3A–C and exemplified for the cell line THP-1 in Figure 3A). However, expression of these genes was not or only slightly induced in the AraC-Res cell lines (Figure 3A and Supplementary Figure S3A–C). Furthermore, the amount and/or activity of autophagosomes and lysosomes clearly increased upon AraC treatment of all parental cell lines, as visualized using fluorescent microscopy (Figure 3B,C) and quantified as a fold change in signal intensity using flow cytometry (Figure 3D,E). In contrast, there was no increase in autophagosomes and lysosomes in AraC-Res cell lines upon AraC treatment (Figure 3D,E). Correspondingly, protein levels of LC3B-II clearly increased upon treatment with AraC, especially after 48 h of treatment (Figure 3F). Since increased levels of LC3B-II are also observed upon autophagy inhibition, p62 levels were also determined as the level of this cargo protein decreases during execution of autophagy. In line with autophagy induction, p62 levels decreased upon AraC treatment in three out of four cell lines, having the lowest levels after 48–72 h (Figure 3F), indicating execution of autophagy. In line with this data, the levels of LC3B-II increased in all AraC-treated cell lines upon the incubation with CQ after 48 h (Figure 3G), further confirming autophagy was activated in parental AML cells upon AraC treatment. Thus, although there is no difference in basal autophagic flux between parental and AraC-Res cell lines, AraC-sensitive AML cells increase the activity of the autophagy pathway upon treatment with AraC, whereas AraC-Res cells do not.

### 2.5. Autophagy Inhibitors Increase the Efficacy of AraC in Parental Cells, but Do Not Re-Sensitize AraC Resistant Cells for AraC

**Figure 4:**
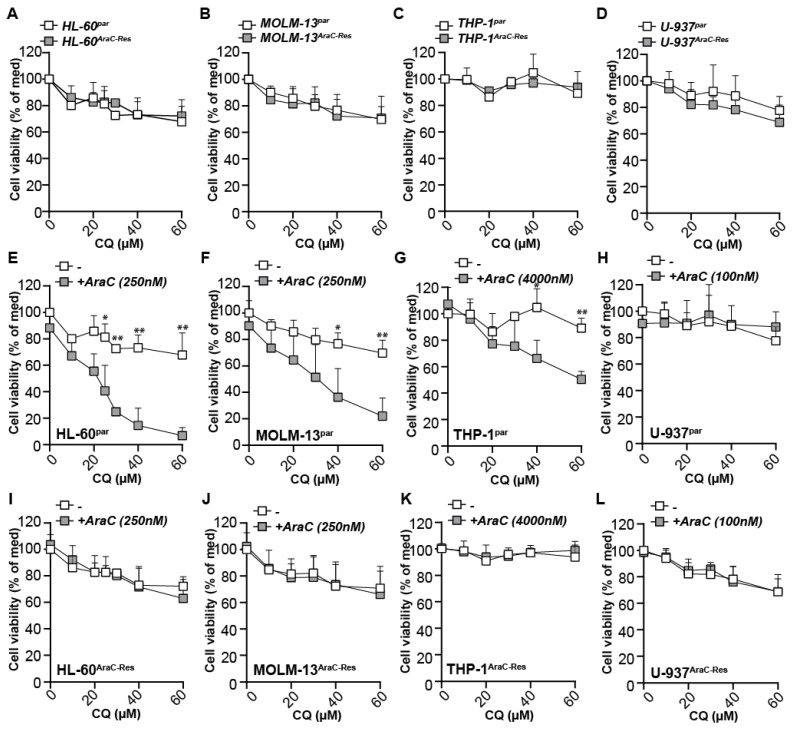
The autophagy inhibitor chloroquine (CQ) increases the efficacy of AraC in parental cells, but does not re-sensitize AraC-resistant cells for AraC. (**A**–**D**) Cell viability (MTS assays) of HL-60, MOLM-13, THP-1, and U-937 cell line pairs upon treatment with a concentration range of CQ (72 h, *n* = 3). (**E**–**H**) Parental HL-60, MOLM-13, THP-1, and U-937 cells were treated for 72 h with various concentrations of CQ in combination with a sublethal dose of AraC. Cell viability was measured using MTS assays after 72 h of incubation (at least *n* = 3) (**I**–**L**) As in (**E**–**H**) but using the AraC-Res cell lines. Of note, CQ was pre-incubated for 1 h before adding AraC. Significance was tested using student’s *t*-test. *p* values are indicated as: ** *p* < 0.01, and * *p* < 0.05.

Corresponding to the equal levels of autophagic flux between parental and AraC-Res cell lines, long-term treatment (72 h) with CQ had equal effects on cell viability in all cell line pairs (Figure 4A–D). Correspondingly, EC50 values were comparable between parental and AraC-Res cells in three out of four cell line pairs, where only THP-1^AraC-Res^ cells were a bit more sensitive for CQ compared to parental cells, but only at one of the tested concentrations (Supplementary Figure S4A–D). Thus, intrinsic sensitivity to this autophagy inhibitor was not altered in the cell pairs. Nevertheless, autophagy inhibitors have previously been postulated to help to overcome AraC resistance. Therefore, treatment with AraC was combined with CQ, as this drug has been FDA-approved and can be easily incorporated into clinical practice. As expected, CQ strongly and dose-dependently increased the efficacy of AraC treatment in three out of four parental cell lines (Figure 4E–H). In contrast, CQ did not impact on AraC efficacy at all in any of the AraC-Res cell lines, even at the highest dose tested (Figure 4I–L). Of note, in these experiments, there was no difference in sensitivity of CQ alone between parental and AraC-Res cells (Figure 4A–D). In the U-937 cell line pair, the addition of CQ did not impact on AraC sensitivity in either parental nor AraC-Res cells. In this cell line, increasing the dose of AraC in combination with a fixed dose of CQ also did not lead to additive effects (Supplementary Figure S4E,F). Similarly, a fixed dose of CQ in combination with a dose range of AraC increased the induction of cell death at a comparable level for all AraC doses in HL-60 parental but not AraC-Res cells (Supplementary Figure S4G,H), suggesting that the optimal concentrations for additive effects can be best determined by varying the dose of CQ.

**Figure 5:**
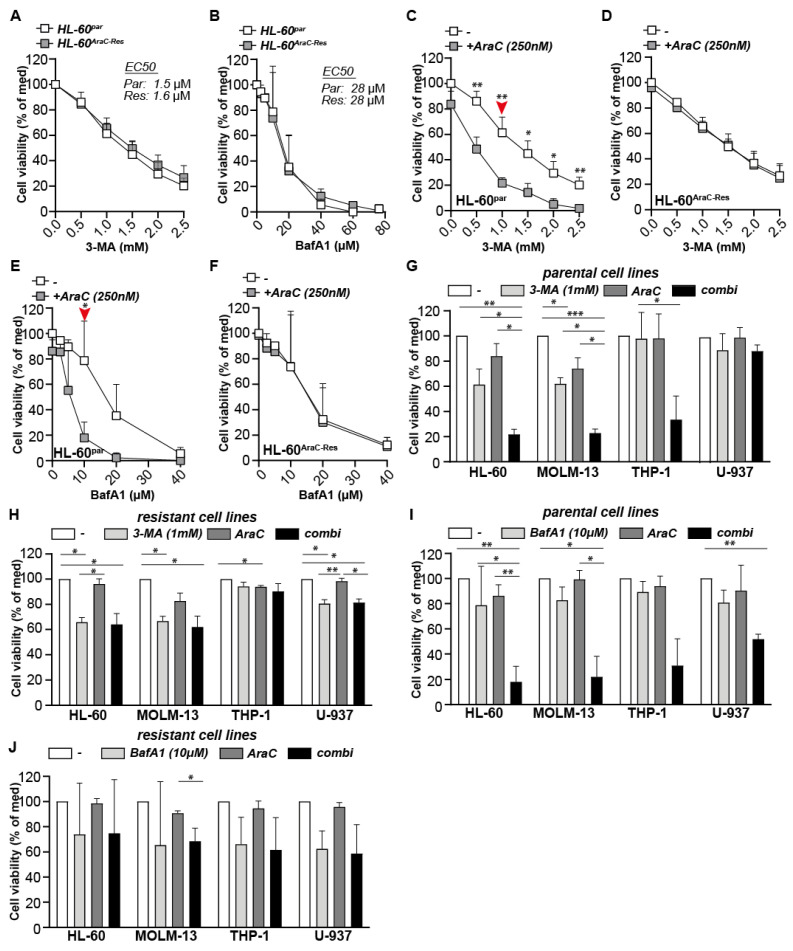
Autophagy inhibitors 3-Methyladenine (3-MA) and Bafilomycin-A1 (BafA1) increase the efficacy of AraC in parental cells, but do not re-sensitize AraC resistant cells for AraC. (**A**,**B**) Cell viability of HL60^Par^ and HL60^AraC-Res^ cells upon treatment with a concentration range of 3-MA and BafA1 (MTS assay after 72 h of incubation, *n* = 3) (**C**–**F**). Cell viability (MTS assay) of HL60^Par^ versus HL60^AraC-Res^ cells upon treatment with various concentrations of 3-MA or BafA1 in combination with a sublethal dose of AraC (250 nM, 72 h, *n* = 3). The red arrows indicate the optimal 3-MA and BafA1 concentration to observe additive effects in combination with AraC, which is used in (**G**–**J**). (**G**,**H**) All parental cell lines (**G**) and AraC-Res cell lines (**H**) treated with a combination of AraC (sublethal dose) and 3-MA (1 mM) and evaluated for cell viability (72 h incubation) using MTS assays (*n* = 3). (**I**,**J**) All parental cell lines (**I**) and AraC-Res cell lines (**J**) treated with a combination of AraC (sublethal dose) and 3-MA (1 mM) and evaluated for cell viability (72-h incubation) using MTS assays (*n* = 3). Significance was tested using students *t*-test (**A**–**F**) or one-way ANOVA Tukey’s multiple comparison test (**H**–**J**). *p* values are indicated as: *** *p* < 0.001, ** *p* < 0.01, and * *p* < 0.05.

Although CQ is the first choice autophagy inhibitor, certain studies reported that CQ may have cytotoxic effects unrelated to autophagy [13]. Therefore, two other autophagy inhibitors, 3-Methyladenine (3-MA) and Bafilomycin-A1 (BafA1), were tested as well. Of note, 3-MA inhibits the autophagy pathway at the level of autophagosome formation via the inhibition of type III Phosphatidylinositol 3-kinases (PI-3K), whereas BafA1 inhibits autophagy at the same level as CQ, i.e., at the fusion of autophagosomes with lysosomes. Both CQ and BafA1 strongly accumulated LC3B-II and p62 in parental HL-60 cells after 24 h and 72 h of incubation (Supplementary Figure S4I,J,L), indicative of autophagy inhibition at the level of autophagosome-lysosome fusion. Also 3-MA functioned as it should, by reducing the amount of LC3B-I, which is indicative of a reduced initiation of autophagosome formation (Supplementary Figure S4I,K). As shown for the HL-60 cell pair, both 3-MA and BafA1 were equally toxic for parental and AraC-Res cells (Figure 5A,B). Furthermore, 3-MA as well as BafA1 sensitized parental HL-60 cells for AraC treatment (Figure 5C,E), whereas both autophagy inhibitors did not improve the efficacy of AraC on AraC-Res cells (Figure 5D,F). At optimal inhibitor concentration levels (as indicated with the red arrow in Figure 5C,E), the effect of 3-MA and BafA1 on the other cell line panels was determined. Like HL-60, 3-MA also increased the induction of cell death by AraC in parental MOLM-13 and THP-1 cells, but not in AraC-Res cells (Figure 5G,H), as in the latter case, where all cell death in the combi was equal to 3-MA alone. As seen for CQ, 3-MA did not impact on the sensitivity of neither parental nor AraC-resistant U-937 cells for AraC, as there was no significant difference between AraC alone and the combination with CQ (Figure 5G,H). In contrast to CQ and 3-MA, BafA1 sensitized all parental cell lines for AraC treatment (Figure 5I), but again did not impact the effect of AraC on AraC-Res cell lines (Figure 5J). Thus, the inhibition of autophagy sensitizes parental AML cells for AraC treatment, whereas it does not re-sensitize AraC-Res cells.

### 2.6. The Combination of CQ and AraC Is Most Effective in Inducing Cytotoxic Effects in AML Patient Samples, but Does Not Re-Sensitize Relapse AML Samples for AraC

**Figure 6:**
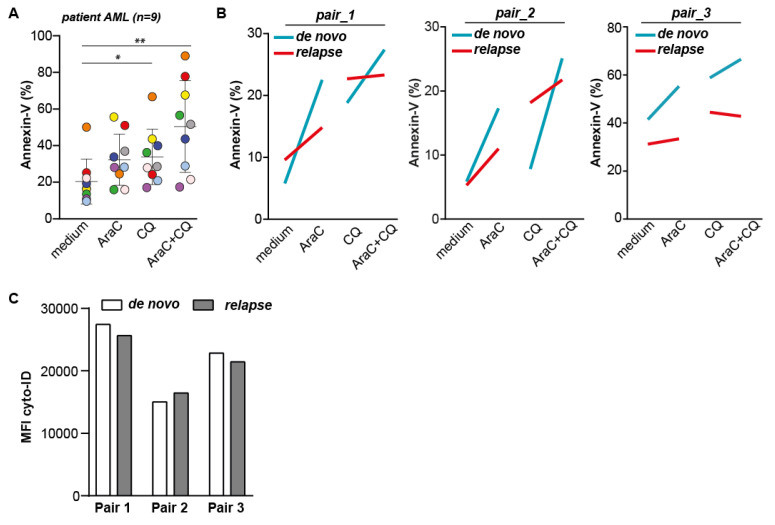
Co-treatment with AraC and chloroquine (CQ) gives the highest levels of cell death in patient-derived AML cells. (**A**) Induction of cell death in AML patient samples (*n* = 9) treated with AraC (750 nM), CQ (10 μM), or the combination as determined by Annexin-V staining after 72 h of incubation. Each color represents a different sample. (**B**) Induction of cell death in de novo versus matched relapse AML patient samples (*n* = 3), treated with 750 nM AraC, 10 µM CQ, or the combination. (**C**) Autophagosomal content as determined by cyto-ID of de novo versus matched relapse AML patient samples (*n* = 3, using flow cytometry). Significance was tested using one-way ANOVA Tukey’s multiple comparison test. *p* values are indicated as: ** *p* < 0.01, and * *p* < 0.05.

Based on the above data with cell lines, AraC sensitive AML cells increase the level of autophagy during AraC treatment and inhibition of autophagy can increase the cytotoxic effect of AraC treatment. In contrast, the inhibition of autophagy did not re-sensitize or increase the effect of AraC in resistant cells, which argues for the timely inhibition of autophagy in AML patients. To study this hypothesis in a more clinically relevant setting, a panel of nine patient-derived AML samples were tested for their response toward AraC in combination with CQ. Treatment with AraC alone yielded an increase in cell death in six out of nine patient samples (Figure 6A), with a maximum response of ~40% additional cell death compared to untreated cells, although not being significant. In addition, treatment with the selected dose of CQ alone yielded a significant increase of ~10–20% cell death compared to the untreated control (Figure 6A). The highest levels of cell death were obtained by the combinational treatment of AraC and CQ, which was significantly increased when compared to untreated samples. However, although in most samples, the combination of AraC and CQ induced the highest level of cell death, this amount was not higher than the sum of each compound alone, suggesting there was no additive effect of the combinational treatment (Figure 6A). Since the data obtained in the cell line panel showed that the inhibition of autophagy only increased the sensitivity for AraC in sensitive parental, but not in AraC-Res cells, we also included three matched AML de novo and relapse patient samples in our study. As expected, de novo patient samples were more sensitive toward AraC treatment compared to relapse samples (~10–20% more cell death), suggesting that relapse samples were less sensitive toward AraC treatment (Figure 6B). However, although the highest levels of cell death were again detected in cells treated with both CQ and AraC, there was no additive effect of the combinational treatment in either de novo or in relapse samples (Figure 6B). Of note, there was also no difference in basal levels of autophagy between de novo and relapse AML patient samples, conforming the cell line data that AraC resistant/less sensitive cells do not have an increased activity of the autophagy pathway (Figure 6C). Thus, the highest levels of cell death are achieved upon co-treatment with AraC and CQ. However, there is no difference in response between de novo and relapse AML samples.

## 3. Discussion

In the current study, we identified that the activity of the autophagy pathway is not elevated in AML cell lines that are stably resistant to AraC treatment. Correspondingly, such AraC-resistant AML cells could not be re-sensitized to AraC using autophagy inhibitors. In contrast, treatment of parental AraC-sensitive cells with AraC did activate autophagy and, correspondingly, the efficacy of AraC was increased by co-treatment with autophagy inhibitors (CQ, 3-MA, and BafA1). However, although inducing the highest levels of cell death, no additive cytotoxic effects of co-treatment with AraC and autophagy inhibitor CQ were detected in a panel of patient-derived AML samples, including 3 matched de novo and relapse samples.

The induction of autophagy upon AraC treatment in AraC-sensitive AML cells as identified here is in line with previous reports [4,14]. In these studies, an increase in the therapeutic effect of AraC was also detected when combining AraC with autophagy inhibitors [4,7]. However, in our study, none of the autophagy inhibitors that were tested (CQ, 3-MA, and BafA1) re-sensitized AraC-Res AML cells for AraC. Correspondingly, no elevated basal autophagic flux was detected in these cells. This data is in apparent contrast to previous reports, where AraC-Res cell lines had a higher autophagic flux compared to parental cells. Furthermore, these cell lines were re-sensitized to AraC treatment using autophagy inhibitors [5,6]. Of note, in both these studies, experiments were performed under serum-free conditions, whereas, in the current study, regular serum-containing culture conditions were used. In one of these studies, the inhibition of autophagy failed to potentiate AraC when adding serum to the culture media [6]. Notably, culturing in serum-free media will induce artificial activation of the autophagy pathway (and, potentially, other stress pathways) that may increase the vulnerability to autophagy inhibitors. Thus, based on our data, autophagy inhibition in regular culture conditions does not re-sensitize AraC-Res cells to AraC treatment. Of note, inhibition of autophagy previously prevented the cytotoxic effect of (low dose) AraC treatment, suggesting that the activation of autophagy is required for cytotoxic activity of AraC [14]. However, in the current study an opposite effect was detected with clear, additive cytotoxicity upon combined AraC and autophagy inhibitor treatment.

In the AML cell line panel, there was a clear and strong additive cytotoxic effect of autophagy inhibitors and AraC, whereas, in ex vivo cultures with AML patient samples, the combination of these drugs also yielded the highest level of cell death while not being additive. Notably, in the clinic, AraC is not used as a single treatment, but combined with anthracyclines, i.e., doxorubicin, idarubicin, or daunorubicin, which may also affect the level of autophagy in AML cells and the sensitivity to autophagy inhibitors. Indeed, doxorubicin, idarubicin, and daunorubicin also activate the autophagy pathway, and inhibition of autophagy increases their cytotoxicity [15,16,17]. Therefore, the effect of autophagy inhibition in AML patients may be different than shown in this study due to the combinational use of various anti-cancer agents. Furthermore, the sensitivity of patient-derived AML cells was tested in ex vivo cultures, which do not take the role of the microenvironment, such as support by stromal cells and bone marrow into account. In this respect, co-culturing of AML cells with stromal cells also increased autophagy and chemoresistance in AML cells, which could be prevented by autophagy inhibition [16]. Furthermore, primary cells may rapidly differentiate and die by apoptosis in the absence of bone marrow support [18,19,20,21], and the use of fresh vs. cryopreserved samples may impact on the levels of autophagy and an experimental outcome. Thus, the exact impact of autophagy inhibition on patient-derived AML warrants further investigation, e.g., in murine models such as the scaffold mouse. Scaffold models are used to maintain the stem cell self-renewal properties and to increase engraftment of patient samples, including all important genetic and risk subgroups [22]. Therefore, this model is of interest to investigate the impact of autophagy inhibition on primary patient-derived AML samples.

Although AraC-resistant AML cells were not re-sensitized by autophagy inhibition, the use of autophagy inhibitors may remain of interest as the co-treatment yielded the highest levels of cell death in all cases. To our knowledge, to date, only one clinical trial using autophagy inhibitors was conducted in AML patients, which had to be terminated due to an inability to accrue (NCT02631252). However, several clinical trials have been conducted in patients with various other types of cancers using CQ or its derivative hydroxychloroquine (HCQ) in dose-limiting toxicity studies alone or in combination with established anti-cancer therapies [as summarized by Reference [14]]. An important outcome of these studies is that the maximum tolerated dose of CQ/HCQ is between 600–1200 mg per day. At this dose, inhibition of autophagy is not achieved in solid tumors [23], but can be detected in peripheral blood mononuclear cells [24,25,26]. Therefore, it is likely that the autophagy pathway can be successfully inhibited in circulating AML cells. Whether autophagy can also be inhibited in the bone marrow niche of AML with these concentrations of CQ is an open question, particularly as CQ has a very poor bio-distribution profile [27]. Furthermore, there is only a small therapeutic window of autophagy inhibition with HCQ between CD34^+^ AML cells and healthy, normal, bone marrow-derived CD34^+^ cells [28]. Thus, CQ is likely not the most optimal autophagy inhibitor to use for the treatment of AML.

Whereas CQ did not increase the sensitivity for AraC in the U-937 cell line, BafA1 did increase AraC efficacy in U-937 cells, and, therefore, may have a broader activity profile that may also be more effective in ex vivo patient samples. In addition, other autophagy inhibitors may be of interest, like Lys05, a CQ analogue that is ten-fold more potent than CQ, and increased AraC sensitivity in AML cells under hypoxia [29]. In addition, the novel autophagy inhibitor ROC-325 potentiated the cytotoxic effect of azacitidine, which is another nucleoside analogue, in AML, both in vitro and in vivo [30]. Furthermore, the stage of autophagy inhibition may be of relevance in order to observe increased therapeutic effects of AraC. In other types of cancer, either early stage (e.g., ULK inhibitors) or late stage (e.g., CQ, BafA1) autophagy inhibitors were optimal to observe additive or synergistic effects in combination with cytotoxic drugs [31,32,33,34]. Thus, it is possible that AML patient cells do respond to other autophagy inhibitors, which is of interest to study in further research.

Of note, in our cell line panel, AraC-res cell lines expressed either less *ENT1* or *dCK* compared to parental cells, which are required for the uptake of AraC into the cell and conversion to the active metabolite of AraC, respectively. Therefore, targeting these proteins may be of more relevance in order to re-sensitize AraC-Res AML cells for AraC. Re-expression of wildtype *ENT1* and *dCK* increased AraC sensitivity in cancer cells [8,35,36]. However, gene therapy is currently still at an early stage. The development of high-throughput gene manipulating using CRISPR-cas9 can be of interest in re-expressing *ENT1* or *dCK* in the future [37]. In addition, other approaches may be used to induce *ENT1* expression. First, in AML, the expression of Fms-related tyrosine kinase 3-internal tandem duplications (FLT3-ITD), a common driver mutation in leukemia, reduces AraC sensitivity by lowering ENT1 levels [38]. In line with this data, treatment with the FLT3 inhibitor PKC412 restored AraC sensitivity by increasing ENT1 expression. Second, total ENT1 expression as well as membrane localization of this protein was induced by MEK inhibitor UO126 on pancreatic cancer cells by inhibiting its lysosomal break-down [39]. In this respect, it is tempting to speculate that CQ and BafA1 that inhibit lysosomal activity also (partly) increase AraC sensitivity by increasing ENT1 expression.

In conclusion, inhibition of autophagy does not re-sensitize AraC-Res AML cells for AraC. Furthermore, the inhibition of autophagy using CQ augments cytotoxicity in AraC sensitive cell lines, even though the impact on patient-derived ex vivo cultures is less pronounced. Thus, inhibition of autophagy may improve AraC efficacy in AML patients, but does not seem warranted for the treatment of AML patients that have relapsed with AraC-resistant disease.

## 4. Material and Methods

### 4.1. Cell Lines and Generation of Cytarabine Resistant Cell Lines

THP-1, HL-60, MOLM-13, and U-937 cell lines were originally purchased from American Type Culture Collection and cultured at 37 °C, in a humidified 5% CO_2_ atmosphere in RPMI (Lonza 12-115F, Basel, Switzerland) supplemented with 10% fetal calf serum (Sigma Aldrich, F7524, St. Louis, MO, USA). Cytarabine resistant cell lines were obtained by culturing cells with a gradually increasing dose of cytarabine (AraC) (Cytarabine Accord 100 mg/mL code: Guj/Drugs/1026, RVG1121666 UR) (see results section). Sensitivity toward AraC was regularly tested using MTS cell viability assays (see below). Of note, when cells became resistant, the AraC pressure was discontinued to prevent any influence on autophagic flux by the presence of this drug. On average, AraC-Res cell lines completely resistant to treatment were generated after ~1 year of selection.

### 4.2. Cytotoxicity Assays, Cytarabine, and Treatment with Autophagy Inhibitors

Cell viability assays were essentially performed as described before using MTS assays [40]. 5 × 10^4^ cells were plated in a 48W plate with 200-µl media and treated with the indicated concentrations of AraC or the autophagy inhibitors chloroquine (CQ, LC3B Antibody Kit for Autophagy, L10382, Invitrogen^TM^, Carlsbad, CA, USA), 3-Methyladenine (3-MA, Calbiochem #189490) or Bafilomycin-A1 (BafA1, Sigma Aldrich, B1793-24G). In case of combination treatment, the autophagy inhibitors were pre-incubated for 1 h to inhibit the autophagy pathway before adding AraC.

After 72 h of incubation at 37 °C, MTS (CellTiter 96^®^ AQueous One Solution Cell Proliferation, G3580, Promega, Madison, WI, USA) was added to the culture medium (10% [*v*/*v*]) and incubated at 37 °C until clear color changes of the untreated cells. A maximum death control was obtained by adding 50 µl ‘dead solution’ (70% ethanol containing 10% triton-X) to untreated cells. MTS readout was performed at OD490nM using the Multi-Scan Sky of Thermo Scientific. The absorbance of the maximum death control was subtracted from all experimental values, and the percentage of viability was calculated as a percentage of the untreated control (OD490 treatment/OD490 untreated control × 100%). EC50 and sigmoidal curve fitting correlation coefficients were calculated using the ED50 Plus v1.0 Excel worksheet developed by Dr. Mario H. Vargas at Instituto Nacional de Enfermedades Respiratorias.

### 4.3. Western Blot-Based Detection of Autophagy Levels

To determine basal levels of autophagic flux in AraC-Res versus parental cell lines, AML cells were plated in a 6W plate (1 × 10^6^ cells in 2 mL RPMI + 10% FCS) and treated with 50 μM CQ for 6 h at 37 °C. Subsequently, cells were harvested, washed with PBS, and lysed in 20 μL of lysis buffer (50 mM Tris, 2 mM EDTA, 2 mM EGTA, 150 mM NaCl, 0.1% SDS, 1% NP-40 substitute, 1 μM Na_3_PO_4_ containing protease inhibitor cocktail, SIGMAFAST™ Protease Inhibitor Tablets, S8820). To determine autophagy levels during AraC treatment, cells were plated in a 6W plate (1 × 10^6^ cells in 2 mL RPMI + 10% FCS) and treated with indicated concentrations of AraC (which gave ~20% reduction in cell viability after 72 h of incubation) and incubated for 24, 48, and 72 h at 37 °C. Subsequently, 50 μM CQ was added and incubated for 2 h after which cells were lysed as described above.

Before Western blot analysis, protein concentrations of the lysates were determined using Bradford protein assays (Pierce™ Coomassie (Bradford) Protein Assay Kit, #23200, Waltham, MA, USA). A total amount of 20-μg protein was loaded and separated using gel electrophoresis (10%, 15%, or 4–15% gradient polyacrylamide gels depending on the target protein) and blotted on PVDF blotting paper (Trans-Blot Turbo RTA Midi 0.2 µm PVDF Transfer Kit, #1704273, Biorad, Hercules, CA, USA). Proteins of interest were detected by incubating with primary antibodies (Santacruz: LAMP1 sc-20011 HRP, p62 sc-28359). Abcam beta-actin AC-15-HRP (Thermo scientific: LC3B from the LC3B Antibody Kit for Autophagy, Thermo scientific, L10382, Waltham, MA, USA) at a dilution of 1:200 for Santacruz and a dilution of 1:1000 for other antibodies in 5% BSA for 16 h at 4 °C. If the primary antibody was not directly HRP-conjugated, staining was followed by the incubation with appropriate secondary HRP-conjugated antibodies at a dilution of 1:2000 (Swine anti-Rabbit-HRP p0217/rabbit anti-mouse-HRP p0260, Dako, Denmark) for 1 h at room temperature. Imaging was performed using a Bio-Rad ChemiDoc™ Imager. Densitometry analysis was performed using ImageJ (ImageJ 1.37c).

### 4.4. RTqPCR Assays

To determine mRNA levels of autophagy related genes (*LC3B*, *SQSTM1*, *ATG5*, *LAMP1*, *LAMP2*) and genes associated with AraC resistance (*ENT1, ENT2, dCK, MDR1*), AML cells were plated in a 6W plate (1 × 10^6^ cells in 2 mL RPMI + 10% FCS) and treated with AraC (concentration yielding ~20% reduction in cell viability after 72 h). After 24 h of incubation, cells were harvested, washed with PBS, and lysed in RLT buffer of the mRNA isolation kit (Qiagen RNeasy plus mini kit #74134) and stored at −80 °C. Subsequently, mRNA was isolated following manufacturers recommendations. cDNA was synthesized using iScript™ cDNA Synthesis Kit (Biorad; #1708891), following manufacturer recommendations using the protocol for 1000 ng/20 μL. RTqPCR was performed using SYBRgreen (Biorad, #1725274) and 5 ng cDNA per condition using the thermocycler (Biorad C1000, CFX384 Real Time System) qPCR program: 3 min 95 °C, (5 s 95 °C, 15 s 58 °C → 39 times), 3 s 65 °C, 5 s 95 °C. Primer sequences: *LC3B* (for: TGCGGGCTGAGGAGATACAA, Rev: TCTTTGTTCGAAGGTGCGGC), *SQSTM1* (for: GTGAAGGCCTACCTTCTGGG, Rev: CGTCCTCATCGCGGTAGTG), *ATG5* (For: TGGGATTGCAAAATGATTTGACC, Rev: TCCTAGTGTGTGCAACTGTCC), *LAMP1* (For: ATGTGTTAGTGGCACCCAGG, Rev: TGTTCACAGCGTGTCTCTCC). *LAMP2* (For: TGGCTCCGTTTTCAGCATTG, Rev: TGTCATCATCCAGCGAACACT), *ENT1* (For: ACTCCAAAGTCTCAGCAGCAGG, Rev: TGGTGATGGTGTTCTCGGTTT), *ENT2* (For: CATCAACTCCTTCAGTGCAG, Rev: GAAGAGGGTGCTGTAGGTG), *dCK* (For: GGACACTGAAAACCAACTTCG, Rev: TGCCTGTAGTCTTCAGCAAGAT) and *MDR1* (primerset 1) (For: AGATAAAAGAGAGGTGCAACGG, Rev: TCCTCGAGAAACTGCGAAACA) *MDR1* (primerset 2) (For: CCTGTGAAGAGTAGAACATGAAGAA, Rev: GCACCTCTCTTTTATCTGGTTGC) *MDR1* (primer set 3) (For: TTGCTGCTTACATTCAGGTTTCA, Rev: AGCCTATCTCCTGTCGCATTA).

### 4.5. Fluorescent Microscopy and Flow Cytometry

To determine autophagosomal and lysosomal content in the cell lines, autophagosomes were stained using the CYTO-ID^®^ Autophagy detection kit (Enzo, ENZ-51031-K200, Farmingdale, NY, USA) and lysosomes were stained using lysotracker red DND-99 (Invitrogen, L7528, Carlsbad, CA, USA). In brief, 200 μL of cyto-ID work solution (1:1000 dilution in PBS) or 2 μL of lysotracker work solution (1:10 dilution in PBS) was added to 200 μL cell suspension (containing 50.000 cells), and incubated for 30 min at 37 °C. Subsequently, cells were washed with PBS and analyzed using flow cytometry plus accessory software (BD Accuri^TM^ C6 cytometer, BD Biosciences, San Jose, CA, USA). For fluorescent images, stained cells were imaged using the EVOS Cell Imaging Systems (Thermo scientific, EVOS-FL).

### 4.6. AML Patient Samples

Peripheral blood (PB) and bone marrow (BM) samples of AML patients were studied after informed consent and protocol approval by the Medical Ethical committee of the UMCG in accordance with the Declaration of Helsinki (protocol code NL43844.042.13, 6 January 2014). Mononuclear cells (MNCs) were isolated by ficoll separation and cryopreserved.

### 4.7. Ex Vivo Culturing of Patient-Derived AML Samples

Cryovials of AML patients were thawed and put in pre-warmed newborn calf serum (NCS, Gibco), and centrifuged for 5 min at 450 g. Thereafter, the pellet was re-suspended in pre-warmed NCS mix (4 µM magnesium sulphate (Sigma-Aldrich), 20 U/mL DNase (Roche, Basel, Switzerland), and 5 U/mL Heparin (Pharmacy of the UMCG) and incubated in a 37° water bath for 15 min. After incubation, cells were centrifuged for 5 min at 450 g. The patient-derived AML cells were cultured in Gartners medium (Alpha-MEM, 12.5% FCS, 12.5% horse serum, 50 µM beta-mercaptoethanol (Sigma-Aldrich), 1 µM hydrocortisone (Sigma-Aldrich), and 1% pen-strep (Sigma-Aldrich)) supplemented with G-CSF, IL-3, and N-pate (20 ng/mL of each cytokine), and cultured on a MS5 layer grown on gelatin coated flasks for 2–3 days, after the thawing procedure.

A total of 50,000 AML cells were plated in a 48W plate with a total volume of 200-uL Gartners medium. Cells were treated with different concentration of AraC, CQ, and the combination of both treatments. After 72 h, samples were stained with Annexin V and analyzed using flow cytometer Cytoflex (Beckman Coulter, Brea, CA, USA).

### 4.8. Statistical Analysis

Significance was tested using a student t-test or an one-way ANOVA plus Tukey’s multiple comparison test using Graphpad Prism software. *p* values are indicated as: **** *p* < 0.0001, *** *p* < 0.001, ** *p* < 0.01, and * *p* < 0.05.

## Data Availability

The data presented in this study are available in article and supplementary material.

## Supplementary Materials

The following are available online at https://www.mdpi.com/1422-0067/22/5/2337/s1.
