# Supplementary material for: Inhibition of Autophagy Does Not Re-Sensitize Acute Myeloid Leukemia Cells Resistant to Cytarabine
Source: Int J Mol Sci. 2021 Feb 26;22(5):2337. doi: 10.3390/ijms22052337 (PMC7956277; doi:10.3390/ijms22052337)
Supplement: Supplementary file 1 [file ijms-22-02337-s001.zip › ijms-1101306-Supplementary/Suppl figure legends_IJMS.docx]

**Suppl. Figure 1. AraC-Res cell lines remain resistant after removing the AraC pressure.** (A-H) Sensitivity for AraC as determined by MTS cell viability assays in parental, AraC-Res cell lines under AraC pressure and AraC-Res cell lines without AraC pressure. U-937 (A-E), HL-60 (F), MOLM-13 (G) and THP-1 (H). Significance was tested using students t-test.

**Suppl. Figure 2. Autophagy resistance in cell lines.** (A-D) mRNA expression of genes involved in autophagy resistance (ENT-1 (A), ENT-2 (B), dCK (C) and BCL-2 (D)) in untreated parental THP-1, MOLM-13, U-937 and HL-60. Significance was tested using students t-test.

**Suppl. Figure 3. Parental but not AraC-Res cell lines increase mRNA expression of autophagy genes upon AraC treatment.** (A-C) mRNA expression levels of autophagy genes (LC3B, SQSTM1, ATG5, LAMP1 and LAMP2) in untreated and AraC treated (24h, sublethal dose) parental and AraC-Res cell lines (n=3). HL-60 250nM (A), MOLM-13 250nM (B), U-937 100nM (C). Significance was tested using students t-test.

**Suppl. Figure 4. Autophagy inhibitors increase the efficacy of AraC in parental cells, but do not re-sensitize AraC resistant cells for AraC.** (A-D) Cell viability (using MTS assays) of HL-60, MOLM-13, THP-1 and U-937 parental versus AraC-Res cell lines upon treatment with various concentrations of CQ (72h) as used in the combination treatment assays (n=3). (E-H) Cell viability (using MTS assays) of U-937 (E-F) and HL-60 (G-H) cell line pairs upon co-treatment with varying doses of AraC and a fixed concentration of CQ (25μM). (I-J) Control experiments to show the functionality of the used autophagy inhibitors. LC3B-I, LC3B-II and p62 levels upon (I) 24h or (J) 72h treatment with CQ (40µM), 3-MA (1mM) and BafA1 (10µM) and the corresponding analysis of expression levels using densitometry of (K) LC3B-I and (L) LC3B-II after 24h of incubation. Of note, the EC50 curves were obtained with a different batch of CQ than used in Figure 4. Significance was tested using students t-test.
