# Supplementary figures and images for: Inhibition of Autophagy Does Not Re-Sensitize Acute Myeloid Leukemia Cells Resistant to Cytarabine
Source: Int J Mol Sci. 2021 Feb 26;22(5):2337. doi: 10.3390/ijms22052337 (PMC7956277; doi:10.3390/ijms22052337)

Supplementary Figure 1

□ parental    ● AraC-Res (AraC pressure)    ○ AraC-Res (med w.o. AraC pressure)

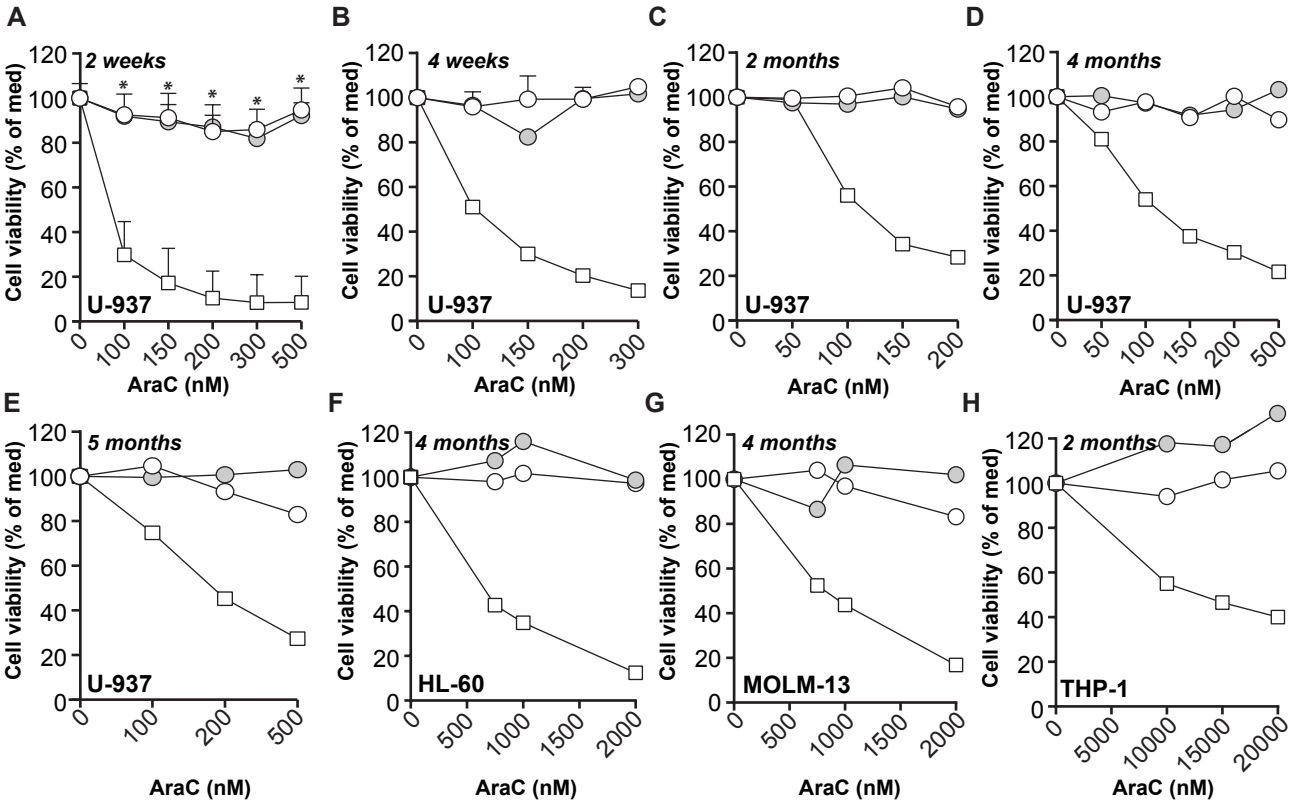

Supplement: Supplementary file 1 [file ijms-22-02337-s001.zip › ijms-1101306-Supplementary/Figure Suppl_1_IJMS_autophagy_new.pdf]

Supplementary Figure 2

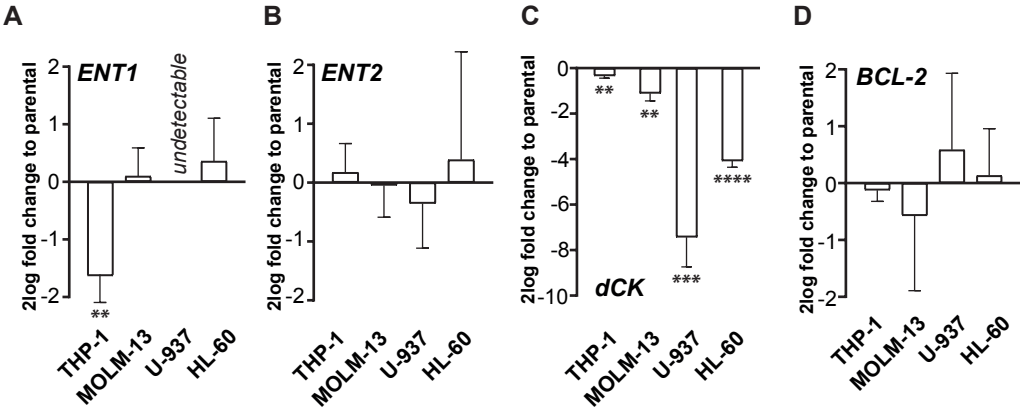

Supplement: Supplementary file 1 [file ijms-22-02337-s001.zip › ijms-1101306-Supplementary/Figure Suppl_2_IJMS_autophagy_new.pdf]

Supplementary Figure 3

A

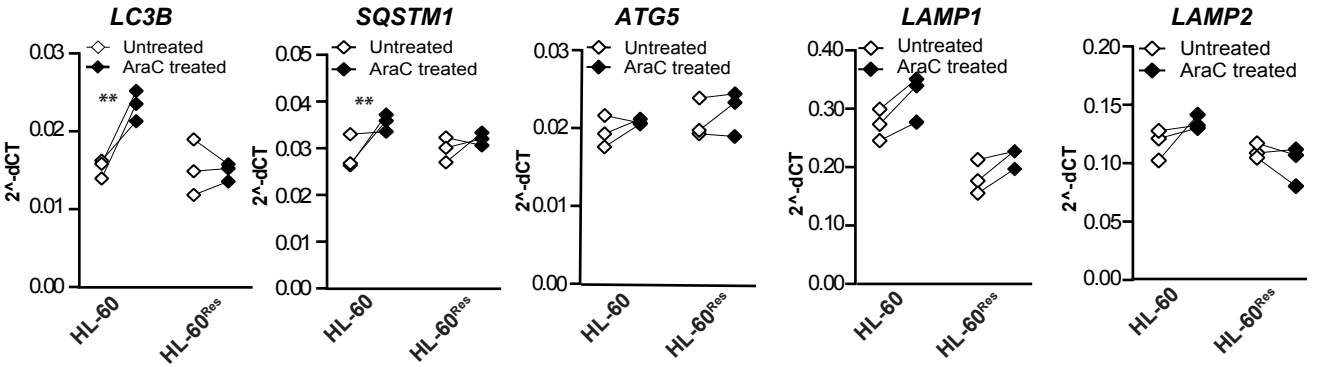

B

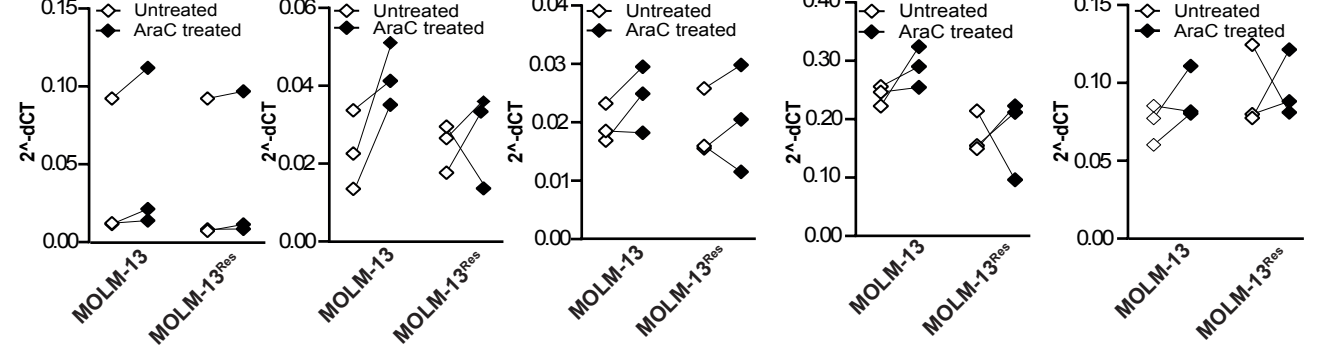

C

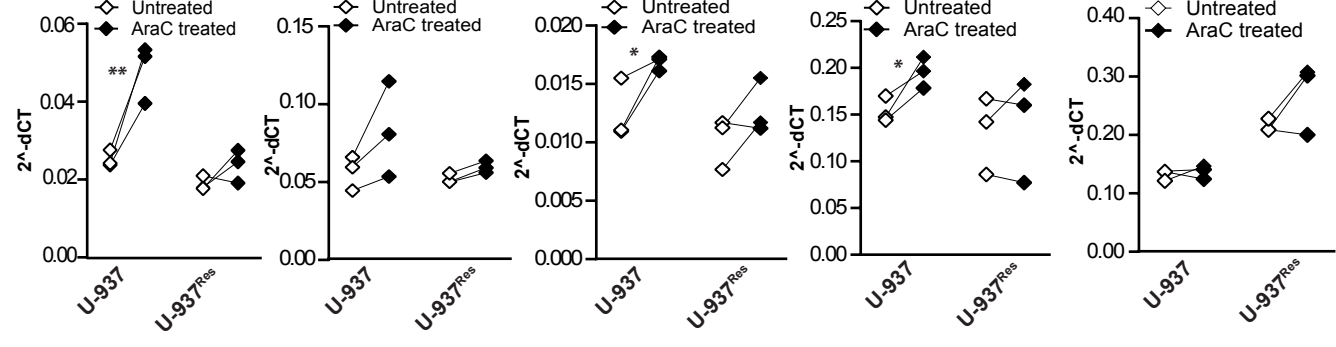

Supplement: Supplementary file 1 [file ijms-22-02337-s001.zip › ijms-1101306-Supplementary/Figure Suppl_3_IJMS_autophagy_new.pdf]

Supplementary Figure 4

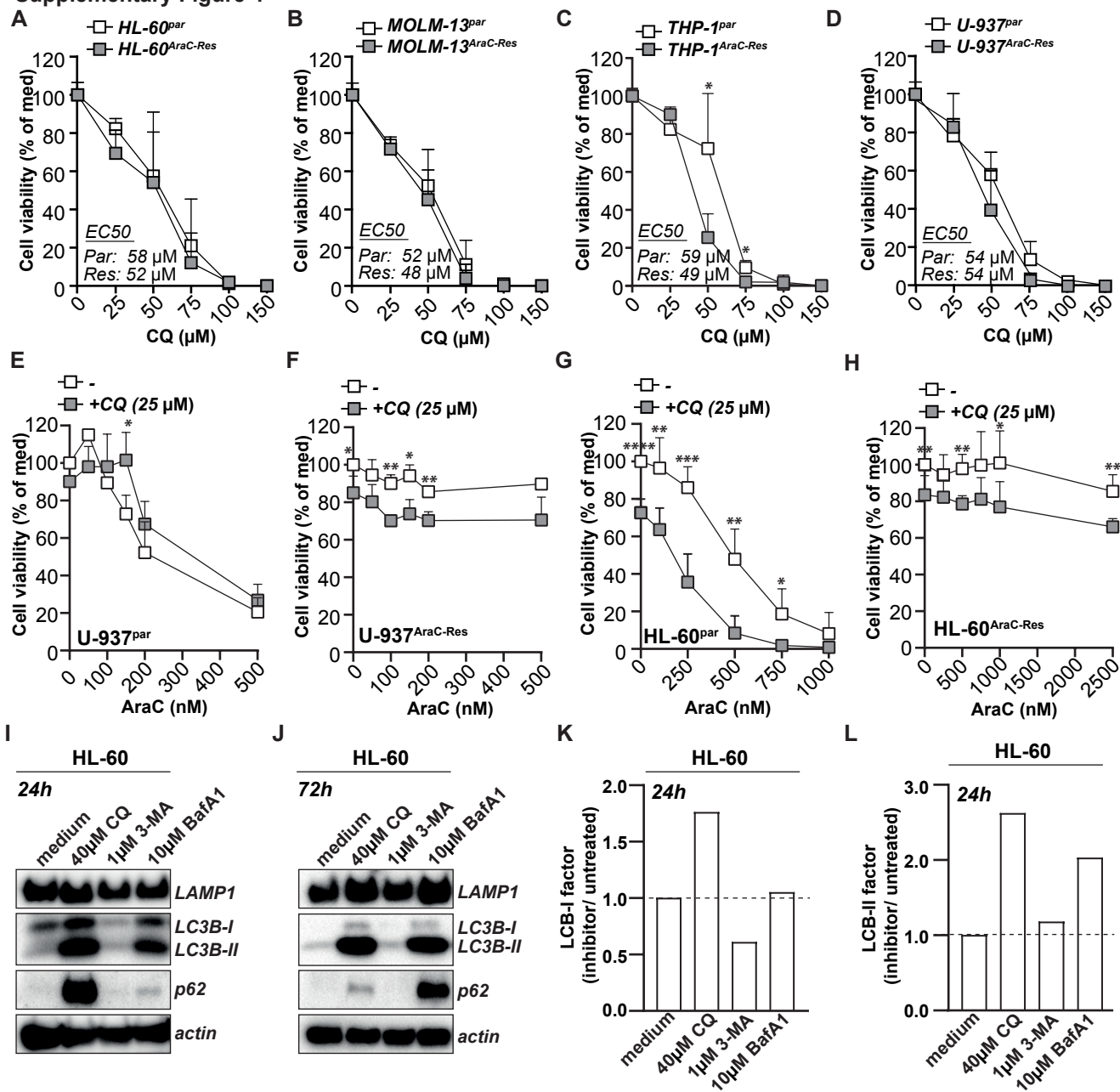

Supplement: Supplementary file 1 [file ijms-22-02337-s001.zip › ijms-1101306-Supplementary/Figure Suppl_4_IJMS_autophagy_new.pdf]
